# Sex‐dependent changes in neuroactive steroid concentrations in the rat brain following acute swim stress

**DOI:** 10.1111/jne.12644

**Published:** 2018-10-07

**Authors:** Ying Sze, Andrew C. Gill, Paula J. Brunton

**Affiliations:** ^1^ Centre for Discovery Brain Sciences University of Edinburgh Edinburgh UK; ^2^ The Roslin Institute University of Edinburgh Edinburgh UK; ^3^ School of Chemistry University of Lincoln Lincoln UK

**Keywords:** 5α‐reductase, glucocorticoids, hypothalamic‐pituitary‐adrenal (HPA) axis, neurosteroids, progestogens, sex differences

## Abstract

Sex differences in hypothalamic‐pituitary‐adrenal (HPA) axis activity are well established in rodents. In addition to glucocorticoids, stress also stimulates the secretion of progesterone and deoxycorticosterone (DOC) from the adrenal gland. Neuroactive steroid metabolites of these precursors can modulate HPA axis function; however, it is not known whether levels of these steroids differ between male and females following stress. In the present study, we aimed to establish whether neuroactive steroid concentrations in the brain display sex‐ and/or region‐specific differences under basal conditions and following exposure to acute stress. Brains were collected from male and female rats killed under nonstress conditions or following exposure to forced swimming. Liquid chromatography‐mass spectrometry was used to quantify eight steroids: corticosterone, DOC, dihydrodeoxycorticosterone (DHDOC), pregnenolone, progesterone, dihydroprogesterone (DHP), allopregnanolone and testosterone in plasma, and in five brain regions (frontal cortex, hypothalamus, hippocampus, amygdala and brainstem). Corticosterone, DOC and progesterone concentrations were significantly greater in the plasma and brain of both sexes following stress; however, the responses in plasma were greater in females compared to males. This sex difference was also observed in the majority of brain regions for DOC and progesterone but not for corticosterone. Despite observing no stress‐induced changes in circulating concentrations of pregnenolone, DHDOC or DHP, concentrations were significantly greater in the brain and this effect was more pronounced in females than males. Basal plasma and brain concentrations of allopregnanolone were significantly higher in females; moreover, stress had a greater impact on central allopregnanolone concentrations in females. Stress had no effect on circulating or brain concentrations of testosterone in males. These data indicate the existence of sex and regional differences in the generation of neuroactive steroids in the brain following acute stress, especially for the 5α‐reduced steroids, and further suggest a sex‐specific expression of steroidogenic enzymes in the brain. Thus, differential neurosteroidogenesis may contribute to sex differences in HPA axis responses to stress.

## 1 INTRODUCTION

Steroid hormones play a crucial role in regulating physiological responses to stress. They can exert their actions in target tissues by binding to classical steroid receptors, which then act as transcription factors in the nucleus to alter gene expression. Additionally, steroids can act in a nonclassical manner. Neuroactive steroids are active metabolites of classical steroid hormones that, independent of their source (ie, peripheral or central), exert rapid nongenomic effects on neuronal excitability by binding to membrane bound ion channel‐linked receptors, thus influencing neurotransmission.^1^ The brain itself can produce neuroactive steroids (referred to as “neurosteroids”), via local de novo synthesis from cholesterol or through the conversion of peripherally derived adrenal or gonadal steroids.^1^, ^2^ In support of this, the brain possesses many steroidogenic enzymes, including 5α‐reductase and 3α‐hydroxysteroid dehydrogenase (3αHSD) which are not only mainly expressed by glial cells,^3^, ^4^, ^5^ but also found in neurones.^6^ Furthermore, neurosteroids continue to be detected in the brain after combined adrenalectomy and gonadectomy,^7^ providing evidence for local neurosteroidogenesis. Nevertheless, the synthesis of neurosteroids is reliant upon the expression of the prerequisite steroidogenic enzymes, which exhibit significant regional differences in the brain.^8^

The major neuroendocrine response to stress is mediated via activation of the hypothalamic‐pituitary‐adrenal (HPA) axis, which results in a net increase in circulating glucocorticoids following activation of corticotrophin‐releasing hormone (CRH) neurones in the medial parvocellular paraventricular nucleus (mpPVN) and adrenocorticotrophic hormone (ACTH) secretion from the anterior pituitary. Glucocorticoids, synthesised in the adrenal cortex, act at multiple targets in the body to facilitate the necessary metabolic and behavioural adaptations required for stress coping and restoring physiological homeostasis. Moreover, glucocorticoids provide negative‐feedback control of the HPA axis through actions on glucocorticoid and mineralocorticoid receptors in the brain and pituitary, crucial for terminating the stress response.

**Figure 1:**
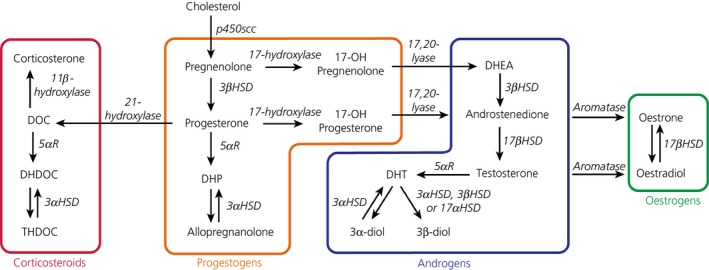
Biosynthetic pathways involved in steroidogenesis. The major pathways and enzymes involved in the biosynthesis of corticosteroids, progestogens, androgens and oestrogens from cholesterol. 3α‐diol, 3α‐androstanediol; 3αHSD, 3α‐hydroxysteroid dehydrogenase (*Akr1c*); 3β‐diol, 3β‐androstanediol; 3‐βHSD, 3β‐hydroxysteroid dehydrogenase (*Hsd3b*); 5αR, 5α‐reductase (*Srd5a*); 17αHSD, 17α‐hydroxysteroid dehydrogenase (*Hsd17a*); 17βHSD, 17β‐hydroxysteroid dehydrogenase (*Hsd17b*); DHDOC: 5α‐dihydrodeoxycorticosterone; DHEA, dehydroepiandrosterone; DHP, 5α‐dihydroprogesterone; DHT, 5α‐dihydrotestosterone; DOC, 11‐deoxycorticosterone; p450scc, cholesterol side‐chain cleavage enzyme (*Cyp11a1*); THDOC, tetrahydrodeoxycorticosterone

In addition to glucocorticoids, stress also results in a rapid increase in the secretion of other steroid hormones such as progesterone^9^ and 11‐deoxycorticosterone (DOC)^10^ from the adrenal cortex. In the brain, these steroids can be further metabolised into neuroactive steroids, such as 5α‐dihydroprogesterone (DHP) and 5α‐dihydrodeoxycorticosterone (DHDOC) by 5α‐reductase, which in turn may be converted into allopregnanolone and tetrahydrodeoxycorticosterone (THDOC), respectively, by 3αHSD (Figure 1), which are considered to fine‐tune and aid cessation of the stress response. Indeed, levels of both allopregnanolone and THDOC are increased in the cerebral cortex and hypothalamus following exposure to acute stress^11^ and both have been shown to negatively modulate stress‐induced HPA axis activity in vivo.^12^, ^13^, ^14^, ^15^ Moreover, allopregnanolone prevents the up‐regulation of CRH mRNA in the mpPVN induced by adrenalectomy, attenuates stimulated CRH release from hypothalamic explants and inhibits the firing of mpPVN CRH neurones in vitro^16^, ^17^, while administration of the 5α‐reductase inhibitor, finasteride, leads to heightened HPA axis responses to stress in males and females.^13^, ^18^ Allopregnanolone, DHDOC and THDOC are positive allosteric modulators of GABA_A_ receptors; by prolonging the opening time of chloride ion channels within GABA_A_ receptors, they enhance the inhibitory actions of GABA.^19^, ^20^, ^21^, ^22^ Given that the PVN is substantially innervated by GABAergic neurones^23^, ^24^ and the majority of mpPVN CRH neurones express GABA_A_ receptors,^25^ this provides a means by which local neurosteroids may modulate inhibitory tone over the HPA axis.

It is well established that there are sex differences in both basal and stress‐induced activity of the HPA axis in rodents. Females generally display greater concentrations of circulating corticosterone under nonstress conditions and this is particularly evident at the onset of the dark phase.^26^, ^27^ In addition, the HPA axis is more responsive to both physical and psychological stressors in females, than males, as reflected by greater secretion of ACTH and corticosterone and a larger increase in CRH gene transcription in the mpPVN.^27^, ^28^, ^29^, ^30^, ^31^

Intriguingly, stress‐induced activation of neurones in limbic brain regions, such as the prefrontal cortex and hippocampus, is greater in males than in females.^32^, ^33^, ^34^ Given that these regions project indirectly to the PVN (eg via a relay in the bed nucleus of the stria terminalis) and exert a net inhibitory effect on HPA axis activity,^35^, ^36^, ^37^ lower stress responses in males may reflect greater inhibitory signalling from limbic brain regions to the PVN.

The sex differences in HPA axis activity have been largely attributed to gonadal steroids, given that oestradiol typically has stimulatory actions, whereas androgens have inhibitory effects on HPA axis function. For example, ovariectomy decreases,^38^, ^39^, ^40^ whereas orchidectomy increases,^27^, ^41^, ^42^, ^43^, ^44^, ^45^, ^46^, ^47^ the HPA axis response to stress and these effects of gonadectomy can be normalised with testosterone^41^, ^42^, ^44^, ^46^ or oestradiol^38^ replacement in males and females, respectively. However, the effect of testosterone on HPA axis activity is evidently mediated via the neuroactive metabolite of testosterone, 3β‐androstandiol (3β‐diol).^48^ Likewise, studies indicate that progesterone may counteract some of the stimulatory effects of oestradiol on HPA axis activity,^49^ although this is likely mediated via its 3α‐hydroxy A ring‐reduced neuroactive metabolite, allopregnanolone.^12^, ^13^ Despite these findings, it is not known whether there are sex differences in the stress‐induced neuroactive steroid concentrations in the brain, which could potentially contribute to the differential HPA axis responses in males and females.

Although some studies have quantified the concentrations of a few steroids in the brain in response to foot‐shock, forced swimming or CO_2_ inhalation, previous work has focused on males and measurements have been largely limited to the cerebral cortex or hypothalamus.^11^, ^50^, ^51^, ^52^ In studies where male and females have been directly compared, this has been under nonstress conditions.^53^, ^54^ To date, no study has simultaneously measured stress‐induced concentrations of multiple steroids in several different brain regions. Moreover, to the best of our knowledge, no study has compared changes in central steroid levels in response to stress in male and female rats in the same experiment. Traditional detection methods such as radioimmunoassays incur limitations: cross‐reactivity issues may hinder accuracy, whereas low throughput and low sensitivity makes quantification of a panel of structurally related steroids in discrete brain regions difficult.^55^, ^56^

Accordingly, in the present study, we used a liquid chromatography‐mass spectrometry (LC‐MS) method to quantify a panel of steroids in the plasma, as well as in five separate brain regions known to be involved in regulating the activity of the HPA axis, from male and female rats under nonstress and acute stress conditions. We aimed to establish: (i) which steroids (from our panel) are increased in the brain by acute stress; (ii) whether there are sex and/or regional differences in stress‐induced central steroid concentrations; and (ii) whether circulating steroid concentrations correlate with those found in the brain following stress.

## 2 MATERIALS AND METHODS

### 2.1 Animals

Male and female Sprague‐Dawley rats were bred in the rodent facility at the Roslin Institute. Rats were group housed by sex (four to six females, three or four males per cage) in open‐top cages with ad libitum access to drinking water and a standard rodent diet (Harlan Teklad; Cambridgeshire, UK) and under a 12:12 hour light/dark cycle (lights on 8.00 am) with controlled temperature (22±1˚C) and humidity (58±3%). Experiments were performed in rats aged 21 weeks and were approved by the local Animal Welfare and Ethical Review Body and performed in accordance with the UK Animals (Scientific Procedures) Act 1986 and the European Directive (2010/63/EU).

### 2.2 Stress paradigm

All experiments and tissue collection were performed in male and female (randomly cycling) rats between 10.00 am and 12.30 pm. Forced swimming was used because: (i) it is a robust activator of the HPA axis^57^, ^58^; (ii) sex differences in ACTH and corticosterone secretion have been demonstrated using this stressor^57^; and (iii) it is a combined physical and psychological stressor, and thus activates stress‐responsive nuclei in both the forebrain and hindbrain.^59^ For the stressed groups (n = 7 per group per sex), rats were placed in a glass cylinder (diameter 25 cm; height 50 cm) filled with water (21‐22°C) to a depth of 30 cm and forced to swim for 2 minutes, after which they were gently dried in a towel and returned to their home cage. Thirty minutes after the onset of swimming stress, rats were rapidly transferred to a separate room and killed by conscious decapitation. This time‐point has previously been shown to correspond with peak levels of immediate early gene induction^59^ in the brain and elevated corticosterone secretion,^60^ as well as with the peak in stress‐induced allopregnanolone concentrations in the brain^11^, ^50^ following swim stress. For the control group, rats (n = 7 per group per sex) remained undisturbed in their home cage prior to killing (as before). In each case, trunk blood was collected in tubes containing 0.5 mL of ice‐cold 5% (w/v) EDTA and plasma separated by centrifugation (1500 *g* at 4°C for 15 minutes). Brains were rapidly removed, and the regions of interest (frontal cortex, hypothalamus, amygdala, hippocampus, brainstem) were dissected (see Supporting information, Figure S1) and frozen on dry ice. Plasma was stored at −20°C and brain samples were stored at −80°C until further analysis.

### 2.3 Steroid standards

All steroid standards were purchased from Steraloids Inc (Newport, RI, USA) and all solvents/chemicals used were high‐performance liquid chromatography (HPLC)‐MS grade. Stock solutions of steroid standards (1 mg mL^‐1^) were prepared in methanol (Honeywell Riedel‐de Haën, Seelze, Germany) and stored at −20°C. Steroids were combined and diluted in methanol into a working stock solution containing a concentration of 10 μg mL^‐1^ of each steroid (except for allopregnanolone and progesterone with a concentration of 25 μg mL^‐1^). On the day of sample processing, standards were further diluted 100‐fold in a surrogate matrix 4% (w/v) bovine serum albumin (BSA) (VWR, Leicester, UK) in PBS, followed by a serial 2.5‐fold dilution in 4% BSA to produce seven calibrants. The calibration standards used ranged from 41 to 10 000 pg mL^‐1^ for corticosterone, DOC (4‐pregnen‐21‐ol‐3,20‐dione), DHDOC (5α‐pregnan‐21‐ol‐3,20‐dione), testosterone, pregnenolone and DHP (5α‐pregnane‐3,20‐dione), and 102.4 to 25 000 pg mL^‐1^ for progesterone and allopregnanolone (5α‐pregnan‐3α‐ol‐20‐one). The deuterated internal standard progesterone‐d9 was also dissolved in methanol and diluted to a final concentration of 25 ng mL^‐1^ in 50% methanol.

### 2.4 Sample processing

Samples from all four groups from any given region were processed in the same run, together with seven standard calibrants and a zero sample containing only 4% BSA. For standard calibrants and plasma, 100 μL was used. Frozen brain regions were weighed prior to sample processing (n = 7 per group per sex, except for the frontal cortex, amygdala and hypothalamus where a sample from the female nonstressed group was lost).

#### 2.4.1 Tissue homogenisation

Brain samples were homogenised in 500 μL of methanol containing 1% formic acid (Fisher Scientific, Loughborough, UK). For plasma and standards, 400 μL of methanol containing 1% formic acid was added to 100 μL of plasma/standards and vigorously vortexed. Next, 20 μL of progesterone‐d9 (25 ng mL^‐1^) was added and the homogenates were briefly sonicated. After incubation on dry ice for 30 minutes, the homogenates were centrifuged for 10 minutes (13 000 *g* at 4°C) and the supernatant was decanted into a borosilicate tube and the pellet re‐extracted again with 500 μL of methanol containing 1% formic acid, and then sonicated and centrifuged as described above but without incubation. Supernatants were combined, then diluted with ultrapure water to a final concentration of 30% methanol (see Supporting information, Figure S2).

#### 2.4.2 Solid phase extraction

Steroids in both plasma and brain samples were extracted by solid phase extraction using DSC‐Discovery C18 100 mg columns (#52602‐U; Supelco, Belfont, PA, USA). Columns were activated with 1 mL of methanol and equilibrated with another 1 mL of 30% methanol. Diluted supernatants from homogenates (approximately 3 mL) were then loaded, followed by two 1‐mL washes of 50% methanol. All steps were assisted by centrifugation at 50 *g* (average flow rate of 0.5 mL min^‐1^). Steroids were eluted with 1 mL of 85% methanol by gravity flow. The collected eluate was dried in a vacuum concentrator (Savant SpeedVac; ThermoFisher Scientific, Waltham, MA, USA) overnight (see Supporting information, Figure S2). Dried samples were stored at −20°C until the day of analysis.

#### 2.4.3 Derivatisation

On the day of LC‐MS analysis, 400 μL of freshly prepared derivatisation agent (1 mg mL^‐1^ of Girard's T reagent; #89397; Sigma‐Aldrich, Gillingham, UK; dissolved in methanol containing 0.2% formic acid) was added to the dried samples. After incubation at 37°C for 30 minutes, the reaction was stopped by the addition of 50 μL of 5% ammonium hydroxide (ACROS Organics, Morris Plains, NJ, USA) in methanol. Samples were dried in the SpeedVac then reconstituted in 50 μL of 25 mmol L^‐1^ phosphate buffer (pH 7.4) in 50% methanol to prevent acid hydrolysis of the derivatisation agent (see Supporting information, Figure S2). The sample was transferred to Chromacol vials (ThermoFisher Scientific) for analysis.

### 2.5 LC‐MS/MS analysis

**Figure 2:**
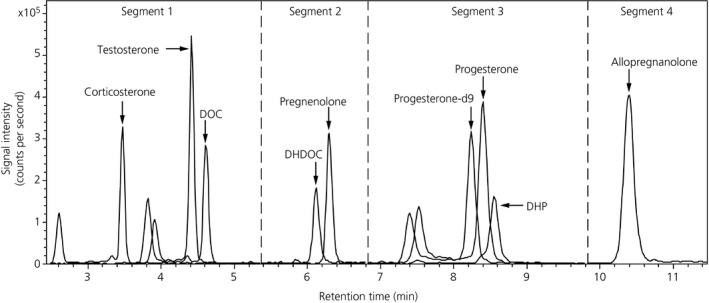
Representative chromatogram of the analytes. For some analytes, a double peak was observed as a result of the presence of both *syn* and *anti*‐Girard's T derivatives, in which case the major product peak was used for quantification. The ratio between the major and minor peak was always consistent between runs. DHDOC: 5α‐dihydrodeoxycorticosterone; DHP, 5α‐dihydroprogesterone; DOC, 11‐deoxycorticosterone

Analysis of steroids was performed using an Ultimate 3000 Dionex HPLC system coupled to an AmaZon ETD ion trap mass spectrometer (Bruker Daltonics, Bremen, Germany). Separation of steroids on reverse phase HPLC was achieved on the ACE UltraCore 2.5 μm Super C18 column (75 mm by 2.1 mm inner diameter; Advance Chromatography Technologies, Aberdeen, UK) maintained at 40°C. Gradient elution was performed (see Supporting information, Table S1) with the mobile phase consisting of 50 mmol L^‐1^ ammonium formate (Thermo Fisher Scientific), pH 3 (solvent A), and methanol with 0.1% formic acid (solvent B). Steroids were analysed simultaneously using multiple reaction monitoring (see Supporting information, Table S2), with positive electrospray ionisation and collision‐induced fragmentation. Injections for all samples, including calibrants, were carried out in duplicate. The method allowed us to simultaneously determine the concentrations of eight steroids: corticosterone, DOC, DHDOC, pregnenolone, progesterone, DHP, allopregnanolone and testosterone (Figure 2) with a total runtime of 19 minutes (see Supporting information, Table S1). Because Girard's T reagent was used as the derivatisation agent, only steroids with a carbonyl group could be examined; thus, oestradiol and the testosterone metabolites, 3α‐ and 3β‐androstanediol, which do not have this functional group, were not detected using this method. THDOC and DHT did not pass the validation criteria and thus these data are not included.

Data were acquired using hystar software (Bruker Daltonics, Bremen, Germany) and peak area under the curve was extracted and automatically integrated using quantanalysis, version 2.0 (Bruker Daltonics). The ratio of the peak area of the target analyte and the peak area of progesterone‐d9 was used to construct the calibration curve. The seven‐point calibration curves were linear for all analytes (see Supporting information, Table S3). Concentrations of samples were extrapolated and converted to ng mL^‐1^ (for plasma) or normalised to the wet weight of the tissues (ng g^‐1^; for brain tissues). Performance characteristics (ie, recovery, sensitivity, accuracy and precision) were determined (see Supporting information, Table S4), and the concentrations of steroids detected in the plasma and brain were consistent with previous studies.^61^, ^62^

### 2.6 Statistical analysis

Data are presented as group means ± SEM. In each case, data were compared using a two‐way ANOVA (sigmaplot, version 11.0; Systat Software Inc., Chicago, IL, USA), with sex and stress as the two main factors followed by Student‐Newman Keuls pairwise multiple comparison testing, except for the testosterone data, which were analysed using a two‐tailed Student's *t*‐test. The relationships between circulating and brain concentrations of steroids were determined using Pearson's correlation coefficients (sigmaplot, version 11.0), and males and females were analysed separately. In each case, *P* ≤ 0.05 was considered statistically significant.

## 3 RESULTS

### 3.1 Corticosterone

#### 3.1.1 Plasma

**Figure 3:**
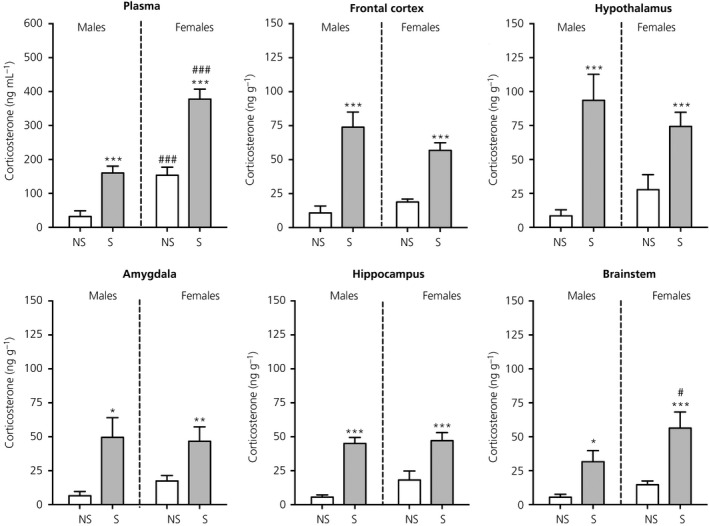
Effect of swim stress on corticosterone concentrations in the plasma and brain. Asterisks denote significant differences in the stressed group (S) compared to the nonstressed group (NS) within the same sex (*P < 0.05; **P < 0.01; ***P < 0.001); hashes denote significant sex differences within the same stress status (^#^ *P* < 0.05; ^###^ *P* < 0.001). Following stress, sex differences were detected for corticosterone concentrations in the plasma and brainstem. n = 6‐7 rats per group. Note the difference in the scale of the *y*‐axis for plasma

As expected, there was a main effect of stress (*F*_1,24_ = 58.9, *P* < 0.001) and sex (*F*_1,24_ = 54.7, *P* < 0.001) on corticosterone secretion and also a significant stress × sex interaction *F*_1,24_ = 4.38, *P* = 0.047). Plasma corticosterone concentrations were significantly greater in the stressed group compared to the control group in both sexes, with females displaying significantly greater plasma corticosterone concentrations compared to males under both stress and nonstress conditions (Figure 3).

#### 3.1.2 Brain

There were no significant differences detected in basal corticosterone concentrations between males and females in any of the brain regions (Figure 3). There were significant main effects of stress in each of the five brain regions examined (frontal cortex, *F*_1,23_ = 50.6, *P* < 0.001; hypothalamus, *F*_1,23_ = 26.7, *P* < 0.001; amygdala, *F*_1,23_ = 14.8, *P* < 0.001; hippocampus, *F*_1,24_ = 45.4, *P* < 0.001; brainstem, *F*_1,24_ = 21.3, *P* < 0.001), with corticosterone concentrations being significantly greater in the stressed rats compared to the nonstressed rats in both sexes (Figure 3). There was a significant main effect of sex observed only in the brainstem (*F*_1,24_ = 5.30, *P* = 0.03), where corticosterone concentrations were 1.8‐fold greater in stressed females compared to stressed males.

#### 3.1.3 Correlations between central and circulating corticosterone

There were significant positive correlations between corticosterone concentrations in the plasma and in all brain regions, except the amygdala in males and the hypothalamus in females (Table 1).

**Table 1:** Correlation between central and circulating steroids

Steroid	Region	Males		Females	
		*r*	*P* value	*R*	*P* value
Corticosterone	Frontal cortex	0.89	<0.001***	0.88	<0.001***
	Hypothalamus	0.81	<0.001***	0.55	0.051*
	Amygdala	0.51	0.062	0.73	0.005**
	Hippocampus	0.82	<0.001***	0.62	0.017*
	Brainstem	0.62	0.019*	0.70	0.006**
DOC	Frontal cortex	0.80	0.001***	0.90	<0.001***
	Hypothalamus	0.76	0.002**	0.75	0.003**
	Amygdala	0.75	0.002**	0.85	<0.001***
	Hippocampus	0.87	<0.001***	0.88	<0.001***
	Brainstem	0.84	<0.001***	0.88	<0.001***
DHDOC	Frontal cortex	−0.07	0.826	0.23	0.459
	Hypothalamus	0.04	0.888	0.60	0.032*
	Amygdala	0.80	0.001***	0.92	<0.001***
	Hippocampus	0.19	0.525	0.68	0.008**
	Brainstem	−0.23	0.427	−0.16	0.591
Pregnenolone	Frontal cortex	−0.010	0.738	−0.02	0.942
	Hypothalamus	0.14	0.631	0.40	0.170
	Amygdala	0.53	0.051*	0.56	0.045*
	Hippocampus	0.06	0.828	0.48	0.079
	Brainstem	−0.25	0.388	−0.15	0.619
Progesterone	Frontal cortex	0.94	<0.001***	0.90	<0.001***
	Hypothalamus	0.98	<0.001***	0.82	0.001***
	Amygdala	0.99	<0.001***	0.83	<0.001***
	Hippocampus	0.94	<0.001***	0.89	<0.001***
	Brainstem	0.93	<0.001***	0.88	<0.001***
DHP	Frontal cortex	0.17	0.571	0.18	0.552
	Hypothalamus	−0.28	0.342	0.50	0.086
	Amygdala	0.83	<0.001***	0.94	<0.001***
	Hippocampus	0.01	0.747	0.66	0.011*
	Brainstem	−0.20	0.491	0.11	0.710
Allopregnanolone	Frontal cortex	−0.03	0.918	−0.02	0.952
	Hypothalamus	0.04	0.907	0.35	0.246
	Amygdala	0.59	0.026*	0.54	0.055
	Hippocampus	0.43	0.127	0.35	0.227
	Brainstem	0.41	0.143	−0.11	0.710
Testosterone	Frontal cortex	0.81	<0.001***		
	Hypothalamus	0.89	<0.001***		
	Amygdala	0.47	0.094		
	Hippocampus	0.49	0.075		
	Brainstem	0.80	0.001***		

Pearson's correlation coefficient (*r*) and probability (*P*) values between plasma and brain concentrations of steroids. Males and females were analysed separately and data from both stressed and nonstressed groups within the same sex were used for analysis (n = 13‐14 per sex).

Asterisks denote significant correlations with plasma concentrations (*P ≤ 0.05; **P ≤ 0.01; ***P ≤ 0.001).

### 3.2 Deoxycorticosterone

#### 3.2.1 Plasma

**Figure 4:**
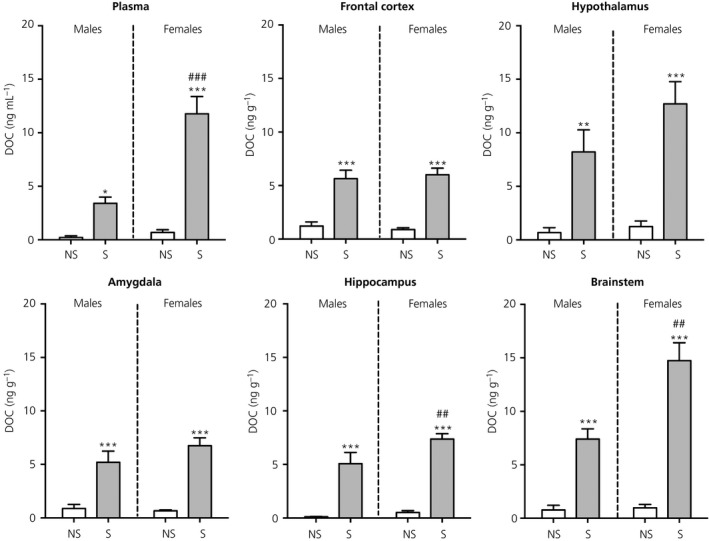
Effect of swim stress on DOC concentrations in the plasma and brain. Asterisks denote significant differences in the stressed group (S) compared to the nonstressed group (NS) within the same sex (*P < 0.05; **P < 0.01; ***P < 0.001); hashes denote significant sex differences with the same stress status (^#^ *P* < 0.05; ^##^ *P* < 0.01; ^###^ *P* < 0.001). After stress exposure, sex differences were detected for DOC concentrations in the plasma, hippocampus and brainstem. n = 6‐7 rats per group

There was no significant difference in plasma DOC concentrations between males and females under nonstress conditions; however, there was a significant effect of stress (*F*_1,24_ = 67.6, *P* < 0.001) and sex (*F*_1,24_ = 26.0, *P* < 0.001) and a significant stress × sex interaction (*F*_1,24_ = 20.7, *P* < 0.001). Stress resulted in significantly greater circulating DOC concentrations in both sexes (Figure 4). However, under stressed conditions, females exhibited significantly greater plasma DOC concentrations compared to males (Figure 4).

#### 3.2.2 Brain

There were no differences in basal concentrations of DOC between males and females in any of the regions studied (Figure 4). There were significant main effects of stress in all five brain regions (frontal cortex: *F*_1,23_ = 72.0, *P* < 0.001; hypothalamus: *F*_1,23_ = 37.1, *P* < 0.001; amygdala: *F*_1,23_ = 56.7, *P* < 0.001; hippocampus: *F*_1,24_ = 103.1, *P* < 0.001; brainstem: *F*_1,24_ = 105.1, *P* < 0.001). Central DOC concentrations were significantly greater in the stressed groups compared to the basal groups for both sexes (Figure 4). There were additional main effects of sex observed for the hippocampus (*F*_1,24_ = 5.34, *P* = 0.03) and brainstem (*F*_1,24_ = 14.4, *P* < 0.001) and a stress × sex interaction in the brainstem (*F*_1,24_ = 12.9, *P* = 0.001). Comparing the stressed groups, females had significantly greater DOC concentrations in the hippocampus and brainstem compared to males but not in the frontal cortex, hypothalamus or amygdala (Figure 4).

#### 3.2.3 Correlations between central and circulating DOC

There were significant positive correlations between DOC concentrations in the plasma and all five brain regions for both sexes (Table 1).

### 3.3 Dihydrodeoxycorticosterone

#### 3.3.1 Plasma

**Figure 5:**
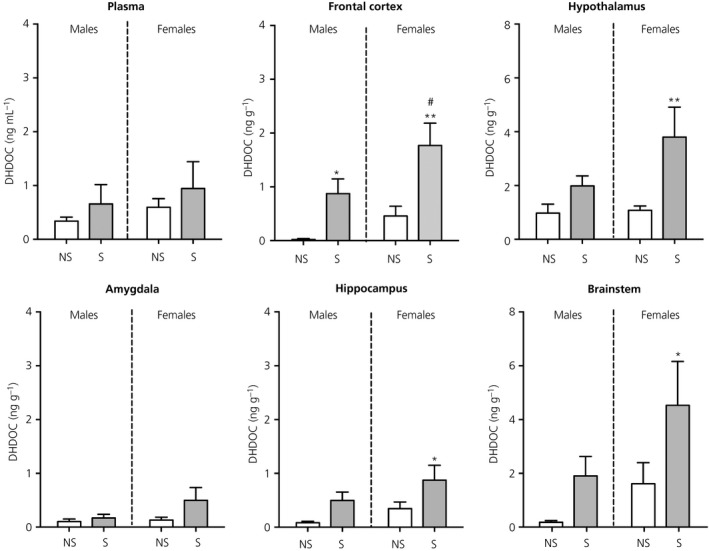
Effect of swim stress on DHDOC concentrations in the plasma and brain. Asterisks denote significant differences in the stressed group (S) compared to the nonstressed group (NS) within the same sex (*P < 0.05; **P < 0.01); hashes denote significant sex differences within the same stress status (^#^ *P* < 0.05). Following stress, there was a significant sex difference in the frontal cortex and a tendency towards greater DHDOC concentrations in the brainstem of stressed females compared to stressed males (*P* = 0.068). n = 6‐7 rats per group. Note the difference in the scaling of the *y*‐axis for the hypothalamus and brainstem

There were no significant differences in plasma DHDOC concentrations in any of the groups (Figure 5).

#### 3.3.2 Brain

There were significant main effects of stress in all brain regions, except for the amygdala (frontal cortex, *F*_1,23_ = 16.1, *P* < 0.001; hypothalamus, *F*_1,23_ = 8.46, *P* = 0.008; hippocampus, *F*_1,24_ = 7.67, *P* = 0.01; brainstem, *F*_1,24_ = 5.68, *P* = 0.025) (Figure 5). There were significant main effects of sex on DHDOC concentrations in the frontal cortex (*F*_1,23_ = 6.09, *P* = 0.02) and brainstem (*F*_1,24_ = 4.35, *P* = 0.048). In the frontal cortex, DHDOC concentrations were greater in both the stressed males and females compared to their respective control groups, with stress‐induced concentrations being significantly greater in females compared to males. However, in the hypothalamus, hippocampus and brainstem, stress resulted in greater DHDOC concentrations only in females.

#### 3.3.3 Correlations between central and circulating DHDOC

In males, only the DHDOC concentration in the amygdala was correlated with plasma levels, whereas, in females, there were positive correlations between DHDOC concentrations in the plasma and those in the amygdala, hippocampus and hypothalamus (Table 1).

### 3.4 Pregnenolone

#### 3.4.1 Plasma

**Figure 6:**
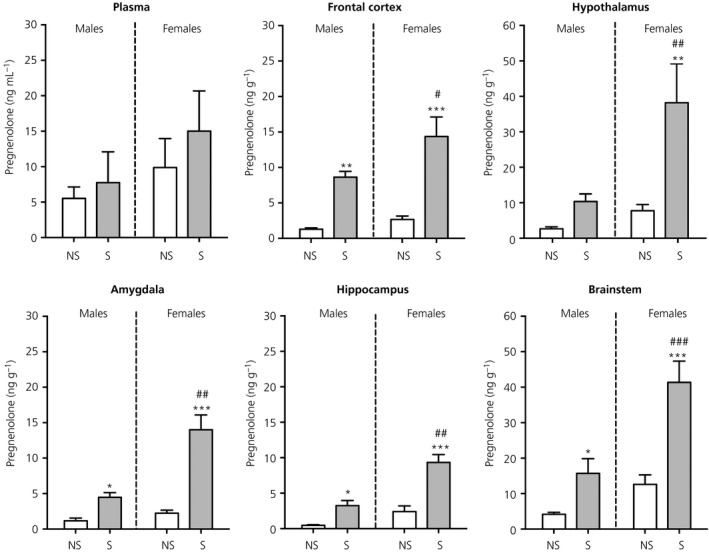
Effect of swim stress on pregnenolone concentrations in the plasma and brain. Asterisks denote significant differences in the stressed group (S) compared to the nonstressed group (NS) within the same sex (*P < 0.05; **P < 0.01; ***P < 0.001); hashes denote significant sex differences with the same stress status (^#^ *P* < 0.05; ^##^ *P* < 0.01; ^###^ *P* < 0.001). Concentrations of pregnenolone were greater in stressed females vs the stressed males in all of the brain regions examined. n = 6‐7 rats per group. Note the difference in the scaling of the *y*‐axis for the hypothalamus and brainstem

There were no significant differences in plasma pregnenolone concentrations between any of the groups (Figure 6).

#### 3.4.2 Brain

There was a significant main effect of sex (frontal cortex, *F*_1,23_ = 5.44, *P* = 0.029; hypothalamus, *F*_1,23_ = 7.75, *P* = 0.01; amygdala, *F*_1,23_ = 20.2, *P* < 0.001; hippocampus *F*_1,24_ = 27.3, *P* < 0.001; brainstem, *F*_1,24_ = 19.1) and stress (frontal cortex, *F*_1,23_ = 39.2, *P* < 0.001; hypothalamus, *F*_1,23_ = 10.4, *P* = 0.004; amygdala, *F*_1,23_ = 40.9, *P* < 0.001; hippocampus; *F*_1,24_ = 39.9, *P* < 0.001; brainstem, *F*_1,24_ = 26.7) in all of the brain regions examined (Figure 6). There was also a significant sex × stress interaction in the brainstem (*F*_1,24_ = 4.89), amygdala (*F*_1,23_ = 12.8, *P* = 0.002) and hippocampus (*F*_1,24_ = 7.25, *P* = 0.01). There was no difference in basal levels of pregnenolone in the brain between the sexes (Figure 6). Following stress, both males and females had greater pregnenolone concentrations than their respective control groups in all brain regions, except for the hypothalamus, where only females showed significantly greater pregnenolone concentrations compared to nonstressed females. For each brain region examined, stress‐induced pregnenolone concentrations were greater in females versus males (Figure 6).

#### 3.4.3 Correlations between central and circulating pregnenolone

For both males and females, the only significant correlation observed with plasma concentrations of pregnenolone was in the amygdala (Table 1). No other significant correlations were detected between circulating and brain pregnenolone concentrations.

### 3.5 Progesterone

#### 3.5.1 Plasma

**Figure 7:**
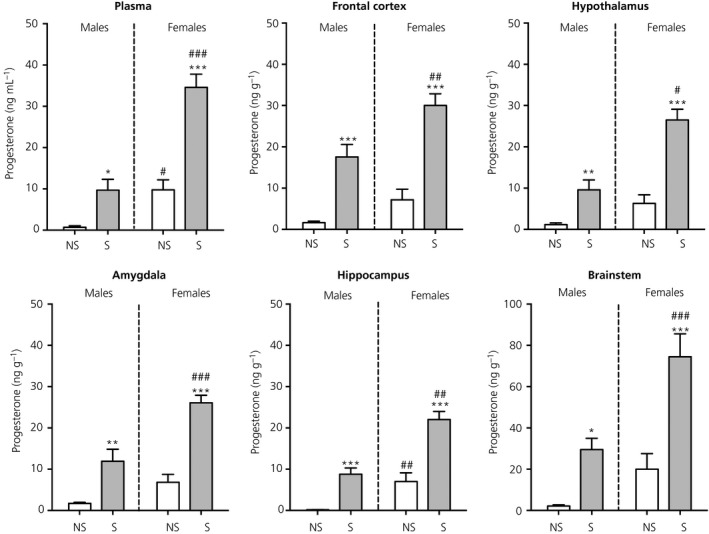
Effect of swim stress on progesterone concentrations in the plasma and brain. Asterisks denote significant differences in the stressed group (S) compared to the nonstressed group (NS) within the same sex (*P < 0.05; **P < 0.01; ***P < 0.001); hashes denote significant sex differences within the same stress status (^#^ *P* < 0.05; ^##^ *P* < 0.01; ^###^ *P* < 0.001). Following stress, sex differences in progesterone concentrations were detected in the plasma and all regions of the brain. n = 6‐7 rats per group. Note the difference in the scaling of the *y*‐axis for the brainstem

There were significant main effects of stress (*F*_1,24_ = 49.1, *P* < 0.001) and sex (*F*_1,24_ = 49.7, *P* < 0.001) and also a significant stress × sex interaction (*F*_1,24_ = 10.8, *P* = 0.003) in plasma progesterone concentrations. Under basal conditions, circulating progesterone was significantly greater in females compared to males (Figure 7). In both sexes, stress resulted in increased progesterone secretion, with greater circulating concentrations in females than in males (Figure 7).

#### 3.5.2 Brain

There were significant main effects of stress and sex in all five brain regions examined (frontal cortex: stress *F*_1,23_ = 62.6, *P* < 0.001 and sex *F*_1,23_ = 13.6, *P* = 0.001; hypothalamus: stress *F*_1,23_ = 48.3, *P* < 0.001 and sex *F*_1,23_ = 28.7, *P* < 0.001; amygdala: stress *F*_1,23_ = 50.7, *P* < 0.001 and sex *F*_1,23_ = 23.0, *P* < 0.001; hippocampus: stress *F*_1,24_ = 53.9, *P* < 0.001 and sex *F*_1,24_ = 38.9, *P* < 0.001; brainstem: stress *F*_1,24_ = 31.9 and sex *F*_1,24_ = 18.8). There were also stress × sex interactions observed for the hypothalamus (*F*_1,23_ = 8.14, *P* = 0.009), amygdala (*F*_1,23_ = 4.48, *P* = 0.045) and hippocampus (*F*_1,24_ = 3.94, *P* = 0.058). There was a tendency for higher basal progesterone concentrations in females compared to males; however, this was only statistically significant in the hippocampus (Figure 7). Following stress, progesterone concentrations in all five brain regions were significantly higher than those measured under basal conditions in both sexes. Moreover, in each case, stress‐induced progesterone concentrations were greater in females than in males (Figure 7).

#### 3.5.3 Correlations between central and circulating progesterone

There were significant positive correlations between circulating progesterone concentrations and those measured in all five brain regions for both males and females (Table 1).

### 3.6 Dihydroprogesterone

#### 3.6.1 Plasma

**Figure 8:**
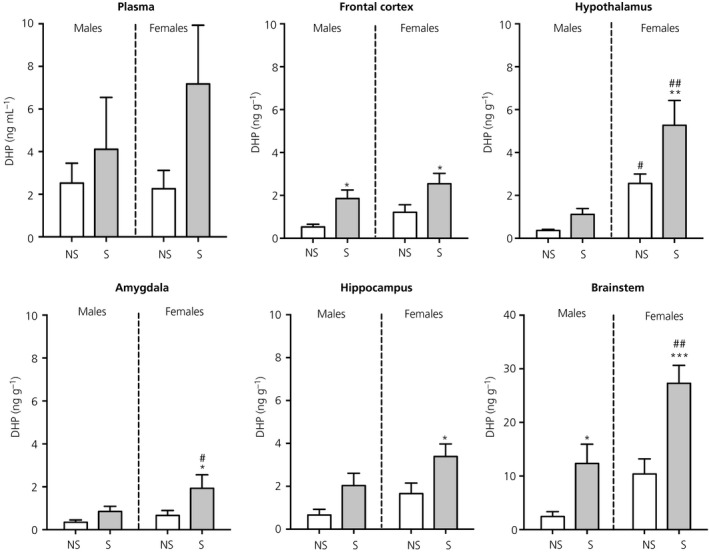
Effect of swim stress on DHP concentrations in the plasma and brain. Asterisks denote significant differences in the stressed group (S) compared to the nonstressed group (NS) within the same sex (*P < 0.05; **P < 0.01; ***P < 0.001); hashes denote significant sex differences within the same stress status (^#^ *P* < 0.05; ^##^ *P* < 0.01). Under nonstress conditions, DHP concentrations were greater in the hypothalamus of females compared to males and a similar trend was detected in the brainstem (*P* = 0.06). Following stress, significant sex differences in DHP concentrations were detected in the hypothalamus, amygdala and brainstem. n = 6‐7 rats per group. Note the difference in the scaling of the *y*‐axis for the brainstem

There was no significant difference in circulating DHP in any of the groups investigated (Figure 8).

#### 3.6.2 Brain

There was a significant main effect of stress on DHP concentration in the frontal cortex (*F*_1,23_ = 13.1, *P* = 0.001), hypothalamus (*F*_1,23_ = 7.14, *P* = 0.01), amygdala (*F*_1,23_ = 5.73, *P* = 0.03), hippocampus (*F*_1,24_ = 9.77, *P* = 0.005) and brainstem (*F*_1,24_ = 22.0, *P* < 0.001), as well as a main effect of sex for the hypothalamus (*F*_1,23_ = 24.0, *P* < 0.001) and brainstem (*F*_1,24_ = 16.0, *P* < 0.001). Under basal conditions, DHP concentrations in the hypothalamus were significantly greater in females than in males and there was a similar trend observed for the brainstem (*P* = 0.06) (Figure 8). Following stress, females had significantly higher DHP in each of the brain regions investigated compared to nonstress conditions, whereas, in males, stress‐induced concentrations of DHP were greater than basal levels only in the frontal cortex and brainstem (Figure 8). There was a sex difference in stress‐induced DHP concentrations, with stressed females exhibiting greater DHP than stressed males in the amygdala, hypothalamus and brainstem (Figure 8).

#### 3.6.3 Correlations between central and circulating DHP

There were significant positive correlations observed between DHP concentrations in the amygdala and circulation in both males and females (Table 1). In females, there was also a modest correlation between the DHP concentrations in the plasma and the hippocampus (Table 1). No other correlations were detected between central and circulating DHP levels.

### 3.7 Allopregnanolone

#### 3.7.1 Plasma

**Figure 9:**
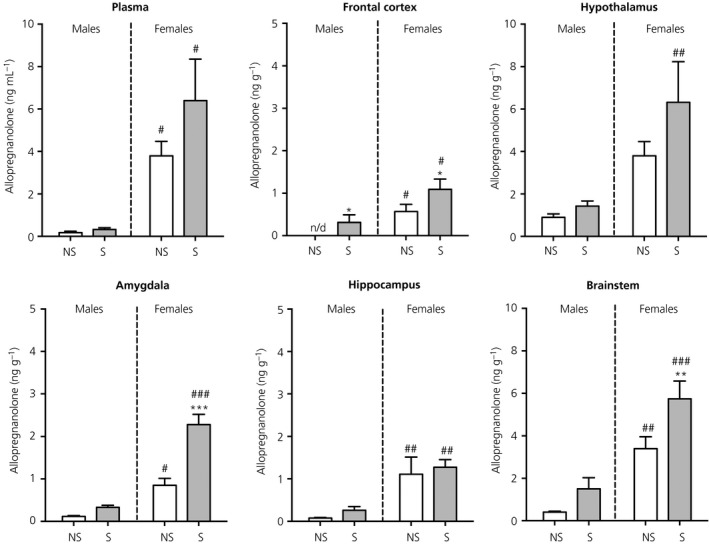
Effect of swim stress on allopregnanolone concentrations in the plasma and brain. Asterisks denote significant differences in the stressed group (S) compared to the nonstressed group (NS) within the same sex (*P < 0.05; **P < 0.01; ***P < 0.001); hashes denote significant sex differences with the same stress status (^#^ *P* < 0.05; ^##^ *P* < 0.01; ^###^ *P* < 0.001). Under nonstress conditions, allopregnanolone concentrations were greater in females compared to males in the plasma and each of the brain regions examined, except the hypothalamus where there was a tendency for higher levels in females (*P* = 0.07). After stress exposure, significant sex differences in allopregnanolone concentrations were detected in the plasma and all regions of the brain. n/d, not detected. n = 6‐7 rats/group. Note the difference in the scaling of the *y*‐axis for the plasma, hypothalamus and brainstem

There was an effect of sex on circulating allopregnanolone concentrations (*F*_1,24_ = 21.9, *P* < 0.001). Basal allopregnanolone concentrations were significantly greater in females than in males (Figure 9). There was no main effect of stress on plasma allopregnanolone concentrations in either sex (Figure 9).

#### 3.7.2 Brain

There were significant main effects of sex on allopregnanolone concentrations for each of the brain regions examined (frontal cortex, *F*_1,23_ = 14.9, *P* < 0.001; hypothalamus, *F*_1,23_ = 13.1, *P* = 0.001; amygdala, *F*_1,23_ = 80.2, *P* < 0.001; hippocampus, *F*_1,24_ = 20.9, *P* < 0.001; brainstem, *F*_1,24_ = 40.2, *P* < 0.001). A significant main effect of stress was observed for allopregnanolone concentrations in the frontal cortex (*F*_1,23_ = 5.71, *P* = 0.03), amygdala (*F*_1,23_ = 27.6, *P* < 0.001) and brainstem (*F*_1,24_ = 9.08, *P* = 0.006). A stress × sex interaction was additionally observed in the amygdala (*F*_1,23_ = 14.8, *P* < 0.001). Basal allopregnanolone concentrations were significantly greater in females than in males in all of the brain regions, except the hypothalamus, where a tendency for higher levels was observed (*P* = 0.07) (Figure 9). Stress resulted in significantly greater allopregnanolone concentrations in the frontal cortex, amygdala and brainstem compared to nonstressed females (Figure 9). Similar trends were observed in the male brain, although this only reached statistical significance for the frontal cortex.

#### 3.7.3 Correlations between central and circulating allopregnanolone

In both sexes, only allopregnanolone concentrations in the amygdala were found to be significantly correlated with circulating allopregnanolone (Table 1).

### 3.8 Testosterone

#### 3.8.1 Plasma

There was no significant effect of stress on plasma testosterone concentrations in males (1.05 ± 0.28 ng mL^‐1^ in nonstressed rats vs 0.77 ± 0.20 ng mL^‐1^ in stressed rats). Testosterone was below the lower limit of quantification (ie <41 pg mL^‐1^) in the plasma from females (see Supporting information, Table S4).

#### 3.8.2 Brain

Testosterone was below the lower limit of quantification in all of the brain regions in females. There was no significant effect of stress on central testosterone concentrations in male rats (frontal cortex, 2.20 ± 0.45 vs 2.20 ± 0.58 ng g^‐1^; hypothalamus, 2.79 ± 0.62 vs 3.15 ± 1.23 ng g^‐1^; amygdala, 0.88 ± 0.17 vs 1.03 ± 0.24 ng g^‐1^; hippocampus, 1.17 ± 0.21 vs 1.71 ± 0.61 ng g^‐1^; brainstem, 2.03 ± 0.19 vs 2.90 ± 0.35 ng g^‐1^ in the stressed and nonstressed groups, respectively).

#### 3.8.3 Correlations between central and circulating testosterone

Concentrations of testosterone in the frontal cortex, amygdala, hypothalamus and brainstem were positively correlated with circulating testosterone in male rats (Table 1).

## 4 DISCUSSION

**Figure 10:**
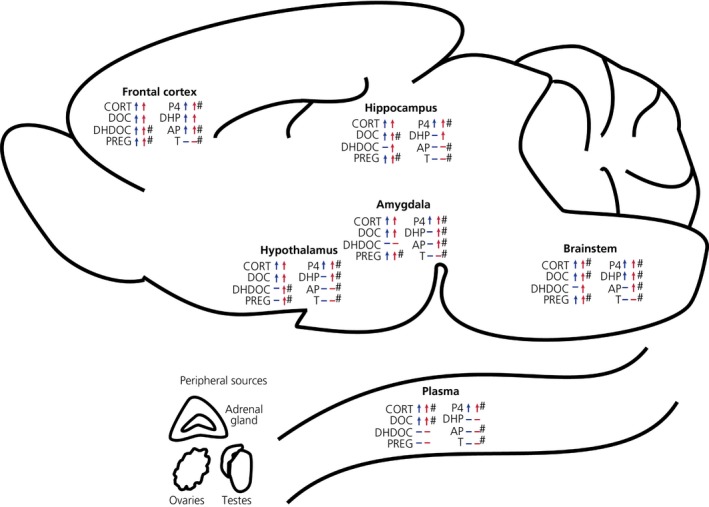
Summary of the key changes in steroid concentrations induced by stress. Changes in steroid concentrations in the plasma and brain 30 minutes after a forced swim stress. The source of steroids in the plasma is largely from peripheral steroidogenic organs, such as the adrenal glands and gonads, whereas steroids in the brain may be peripherally or centrally derived. Blue and red arrows represent stress‐induced increases in steroid concentrations in males and females, respectively. Dashes indicate no significant changes following stress. Hashes denote the presence of a sex difference under stressed conditions. AP, allopregnanolone; CORT, corticosterone; DHDOC, dihydrodeoxycorticosterone; DHP, dihydroprogesterone; DOC, deoxycorticosterone; PREG, pregnenolone; P4, progesterone; T, testosterone

In the present study, we investigated the impact of an acute stressor on the circulating and central steroid profile in male and female rats. Several key findings emerged in the present study. We show for the first time there are clear sex differences in the brain's steroid response to acute swim stress in the rat, with females typically showing markedly greater concentrations in most cases. We also report that, although concentrations of corticosterone, progesterone and DOC in the brain are positively correlated with those measured in the circulation, this is not the case for pregnenolone, DHP, DHDOC and allopregnanolone, supporting the concept of the de novo synthesis of these neurosteroids in the brain in response to stress.^7^, ^11^ Lastly, although the effect of stress on the steroidal milieu of the brain was largely global, we did find evidence for regional differences (Figure 10), particularly for DHDOC and allopregnanolone, which may be of functional relevance.

A key feature of the endocrine response to stress is increased secretion of corticosterone from the adrenal cortex into the bloodstream. It is well established that females display enhanced HPA axis activity, characterised by a greater circulating concentrations of corticosterone under basal conditions and a larger increase in ACTH and corticosterone secretion following acute stress.^63^, ^64^, ^65^ Consistent with this, females in the present study exhibited greater basal and stress‐induced plasma corticosterone concentrations. In the periphery, greater corticosterone secretion in females is associated with a heightened sensitivity of the anterior pituitary to CRH and the adrenal gland to ACTH, as well as a greater capacity for glucocorticoid synthesis in the adrenal glands,^66^ probably as a result of the actions of oestradiol.^39^ In the brain, CRH mRNA expression in the mpPVN is increased following stress to a greater extent in females than in males,^31^ which may be further compounded by diminished GR‐mediated negative‐feedback control of the HPA axis in females.^67^ Interestingly, despite sex differences in the plasma, corticosterone concentrations in the female brain were not different from those in males, except for in the brainstem. This is consistent with the finding of similar circadian corticosterone rhythms and concentrations in the brains of males and females, despite differences in the plasma.^26^, ^27^, ^63^, ^68^ It is likely that greater concentrations of corticosteroid‐binding globulin (which restricts the amount of free corticosterone in the blood that can enter the brain) in females^64^, ^69^ contribute to this finding. It is not known whether sex differences exist in the expression of 11β‐hydroxysteroid dehydrogenase type 1 (which reactivates corticosterone from its inert metabolite, 11‐dehydrocorticosterone) in the brain, which could play a role in modulating the local concentrations of corticosterone in the brain. However, sex and region‐specific differences in 11β‐hydroxylase (Cyp11b1) expression have been reported in the brain,^70^ suggesting that differences in local corticosterone synthesis could also potentially contribute.

Under nonstress conditions, circulating progesterone concentrations were greater in females than males, which is consistent with previous findings^53^, ^71^; however, there was no sex difference in plasma DOC concentrations. Following stress, circulating concentrations of DOC and progesterone were increased in both sexes, with significantly greater responses in females. Stress also resulted in greater levels of progesterone and DOC in both sexes in each of the brain regions investigated. Despite evidence of low levels of 21‐hydroxylase gene expression and activity in the rodent brain,^72^, ^73^ DOC is considered to be primarily synthesised from progesterone in the adrenal cortex and is secreted from the adrenal glands, together with progesterone, in response to stress.^9^, ^10^ Consistent with this was our finding of a positive correlation between circulating DOC and progesterone concentrations and those in the brain, indicating that the likely source of DOC and progesterone in the brain is from the periphery, specifically adrenal derived. Although there was a robust sex difference in circulating DOC after stress, centrally, this was reflected only in the brainstem. This may be a result of sex differences in DOC metabolism in limbic brain regions in females or that circulating DOC more readily accesses the brainstem.

Importantly, DOC and progesterone mediate rapid nongenomic effects indirectly via their downstream neuroactive metabolites THDOC and allopregnanolone (Figure 1), which act as allosteric modulators of GABA_A_ receptor activity^1^, ^74^ to enhance inhibitory GABA neurotransmission. THDOC and allopregnanolone are synthesised from DOC and progesterone via the intermediates DHDOC and DHP, respectively, and their conversion is catalysed by the same enzymes: 5α‐reductase and 3αHSD (Figure 1). In the present study, stress resulted in higher concentrations of both DHDOC and DHP in discrete brain regions but not in the plasma, suggesting de novo synthesis of these steroids in the brain. Moreover, differential regulation was observed depending on sex and brain region. For example, DHDOC concentrations were found to be increased only in the frontal cortex of males following stress, whereas, for females, DHDOC concentrations were increased in the frontal cortex, hypothalamus, hippocampus and brainstem. Similarly, although stress increased DHP concentrations in each of the five brain regions examined in females, in males, stress‐induced elevations in DHP were only detected in the frontal cortex and brainstem. In addition to the presence of greater levels of precursors in the female brain, differential expression of the 5α‐reductase may contribute to the sex difference, given that expression has been shown to be higher in females, at least in the hippocampus.^75^ Moreover, regional differences in expression and activity have been demonstrated for 5α‐reductase and 3α‐HSD in the adult brain,^76^, ^77^, ^78^, ^79^ with sexual dimorphic expression being displayed after stress exposure.^80^ Any direct effects of DHDOC and DHP are unclear, although DHP is known to have affinity for the progesterone receptor^81^ and DHDOC can potentiate GABA‐activated chloride ion currents in neurones,^22^ suggesting that, similar to THDOC and allopregnanolone, DHDOC also acts as a positive allosteric modulator at GABA_A_ receptors.

Roles for allopregnanolone and THDOC in regulating HPA axis activity in response to stress via potentiating the inhibitory effects of GABA are well established.^12^, ^13^, ^14^, ^16^, ^74^, ^82^, ^83^ Unfortunately, we were unable to reliably quantify THDOC using our method; however, we were able to measure allopregnanolone. Under nonstress conditions, circulating and central allopregnanolone concentrations were greater in females than in males, which is consistent with previous findings.^54^, ^71^ We did not observe a significant increase in circulating allopregnanolone concentrations 30 minutes after stress exposure in either sex, in contrast to previous studies in male rats.^11^, ^52^ The reason for this is not clear; however, the different duration/intensity of the swim stress used (2 minutes in the present study versus 10 minutes) may contribute. Moreover, allopregnanolone concentrations are known to peak 30‐60 minutes later in the plasma than in the brain.^11^ Nevertheless, following stress, allopregnanolone concentrations were greater in the frontal cortex, amygdala and brainstem of females compared to controls, as well as in the frontal cortex of males, consistent with previous findings.^11^, ^52^ Sex differences in central allopregnanolone concentrations may result from greater concentrations of the precursors, progesterone and DHP and/or sex differences in the activity of the converting enzymes in the brain. The strong positive correlation between central and peripheral progesterone concentrations, coupled with a lack of correlation between circulating and central allopregnanolone, and its precursor DHP, suggests that DHP and allopregnanolone are synthesised in the brain from progesterone following stress; however, it would be necessary to measure the activity of the converting enzymes in the brain to confirm this. Caruso et al^54^ report a positive correlation between progesterone concentrations in the plasma and cerebral cortex in rats, which is consistent with our findings; however, in contrast to the present study, they also observed a direct correlation between pregnenolone, DHP and allopregnanolone levels in the plasma and cerebral cortex. The reason for this difference is unclear; however, it is important to note that the correlations performed in the present study included data from both stressed and nonstressed rats within the same sex, whereas, in the previous study, correlations were analysed only under basal conditions with data from male and female rats combined. Thus, it is likely that the lack of correlation reported in the present study is a result of stress increasing central levels of pregnenolone, DHP and allopregnanolone, without affecting circulating levels.

It is not clear whether the sex differences in neurosteroids reported in the present study are of functional importance with respect to HPA axis regulation and further studies are needed to establish whether other stressors result in similar effects. Unlike the brainstem, the prefrontal cortex, amygdala and hippocampus do not innervate the PVN directly; rather, they modulate activity of the HPA axis via neural projections to GABAergic relay sites, such as the peri‐PVN, the bed nucleus of the stria terminalis and the medial preoptic area, which exert considerable inhibitory tone over CRH neurones.^24^, ^36^ Thus, it is conceivable that sex differences in brain concentrations of neurosteroids with GABA_A_ receptor agonist activity may lead to differential modulation of upstream inhibitory signalling pathways, resulting, for example, in a greater disinhibition of PVN CRH neurones in females, which could contribute to sex differences in HPA axis responses to stress.

As well as the increase in downstream metabolites of progesterone and DOC, the principal precursor for these steroid hormones, pregnenolone, was also increased throughout the brain following stress, and to a greater extent in the brains of females. This may indicate that, in females, either p450scc is more highly expressed/more active or that pregnenolone is metabolised at a different rate in females; however, this requires further study. Consistent with previous studies, we found no stress‐induced change in plasma pregnenolone concentrations.^52^

One point to note is that the females used in the present study were randomly cycling, which may be expected to influence the steroid concentrations in the brain. However, there is very little variation in brain concentrations of pregnenolone, progesterone, DHP or allopregnanolone across the oestrous cycle when these are measured in the morning (as they were in the present study); indeed, circadian fluctuations in these steroids are far greater than those relating to any stage of the oestrous cycle.^84^ Moreover, oestrous cycle stage does not affect basal corticosterone secretion^85^ or the corticosterone response to swim stress^86^ when sampling is performed in the morning.

The mechanisms involved in stress‐induced increases in neuroactive steroid levels in the brain have yet to be established. One study has indicated that CRH increases concentrations of progesterone, DHP and allopregnanolone in the brain.^87^ Steroidogenic enzymes such as 5α‐reductase are known to be regulated by androgens^88^, ^89^ and, although adrenal‐derived steroids have been shown to increase 3α‐HSD expression in the brain in both sexes, gonadal steroids regulate central 3α‐HSD expression in a sex‐specific manner.^90^ Given that testosterone was unaltered by stress, it is unlikely that it contributes to the rapid changes in stress‐induced neuroactive steroids seen in the present study; however, testosterone may underlie sex differences in steroidogenic enzyme expression in the brain, and therefore influence the brain's capacity to produce neurosteroids.

With respect to rapid regulation of neurosteroid production, GABA may play a role via an ultrashort feedback loop. Brain regions that express neurosteroidogenic enzymes are densely innervated by GABAergic neurones and express abundant GABA_A_ receptors.^91^, ^92^, ^93^ Moreover, GABA or GABA_A_ receptor agonists inhibit the de novo synthesis of neurosteroids^94^ and neurosteroidogenic neurones are evidently under tonic inhibition by GABA.^94^ Given that some neuroactive steroids, such as allopregnanolone, are potent allosteric modulators of GABA_A_ receptor function,^19^, ^20^, ^21^ it is conceivable that neurosteroids may control their own production by regulating the activity of GABA_A_ receptors. Whether such a mechanism contributes to the sex differences in central neurosteroid concentrations requires further study; however, GABA receptor subunit expression does differ between males and females, under basal conditions and following stress.^95^

In summary, the data obtained in the present study demonstrate robust sex differences in the steroid response to acute swim stress and indicate sex‐specific expression of steroidogenic enzymes in the brain. Taken together, our data also support a role for the de novo synthesis of neurosteroids by the brain following stress, particularly those derived from 5α‐reductase activity (ie, DHDOC, DHP, allopregnanolone). The neurosteroid response to stress is likely to be adaptive and disparate neurosteroidogenesis may contribute to sex differences in HPA axis function; however, it is unclear whether greater neuroactive steroid levels in females confer an overall advantage or disadvantage. Stress‐induced increases in neuroactive steroids are likely to have important neuroendocrine and/or behavioural effects to aid stress adaptation. For example, in addition to modulating HPA axis activity, allopregnanolone is known to have anxiolytic and analgesic actions.^82^, ^96^ Sex differences in the brain's response to stress may account for differences in the propensity to develop stress‐related disorders between males and females, further highlighting the importance of considering sex when developing therapies.

## CONFLICT OF INTERESTS

The authors declare that they have no conflicts of interest.
