# Supplementary material for: Sex‐dependent changes in neuroactive steroid concentrations in the rat brain following acute swim stress
Source: J Neuroendocrinol. 2018 Oct 7;30(11):e12644. doi: 10.1111/jne.12644 (PMC6221110; doi:10.1111/jne.12644)
Supplement: Supplementary file 1 [file JNE-30-na-s001.pdf]

SUPPORTING INFORMATION

Sex-dependent changes in neuroactive steroid concentrations in the rat brain following acute swim stress

Ying Sze[1,2], Andrew C. Gill[1,3] & Paula J. Brunton[1,2]*

[1]The Roslin Institute; and [2]Centre for Discovery Brain Sciences, University of Edinburgh, UK; [3]School of Chemistry, University of Lincoln, UK.

*Corresponding author:
Centre for Discovery Brain Sciences, Hugh Robson Building, George Square, Edinburgh, EH8 9XD, UK
Email: p.j.brunton@ed.ac.uk
Telephone: +44 (0) 131 651 1507

No. of figures: 2 No. of tables: 4

Supplementary Figure 1

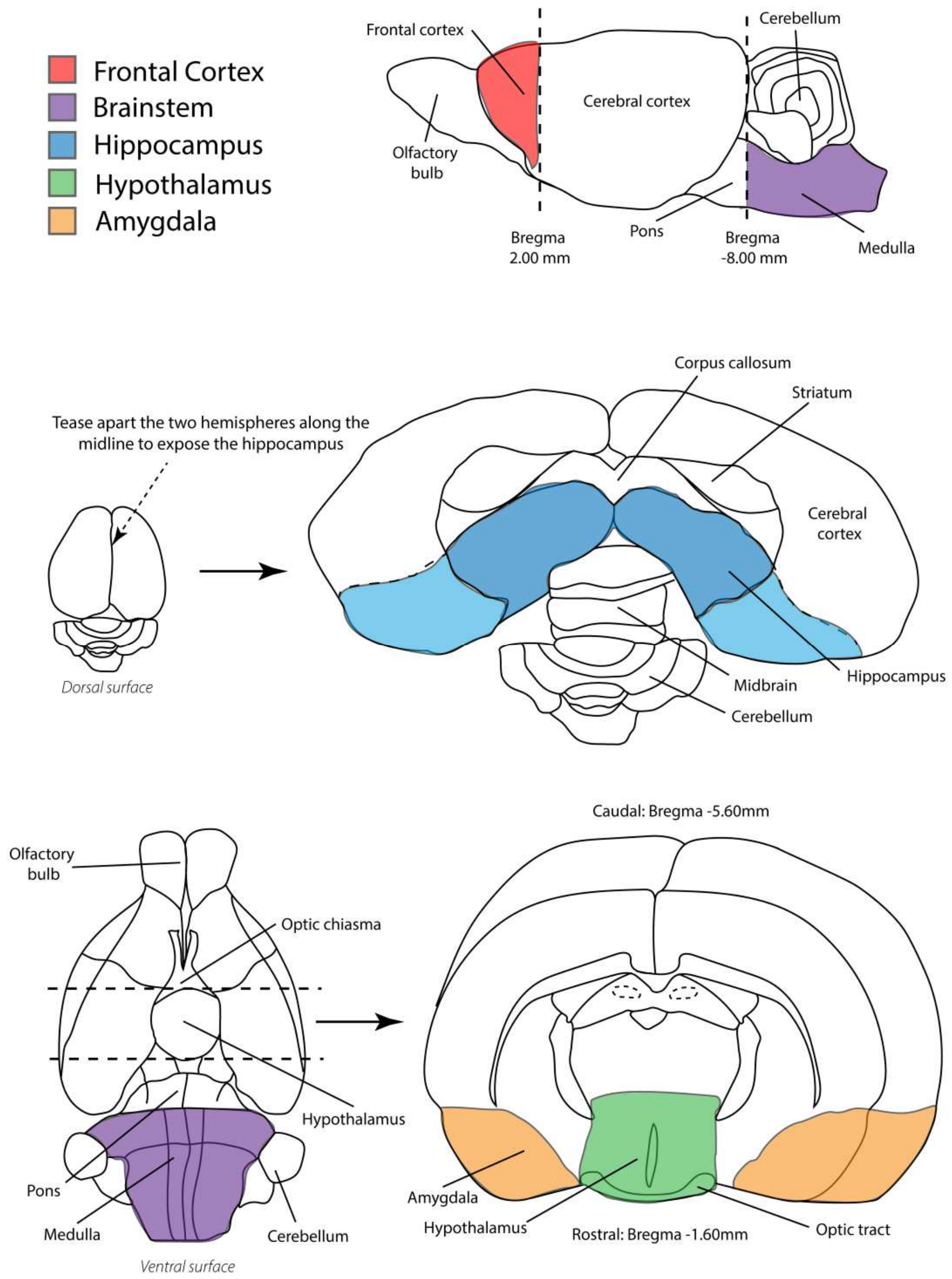

Supplementary Figure 1: Schematic illustrating landmarks used for gross dissection of the different brain regions of interest.

Supplementary Figure 2

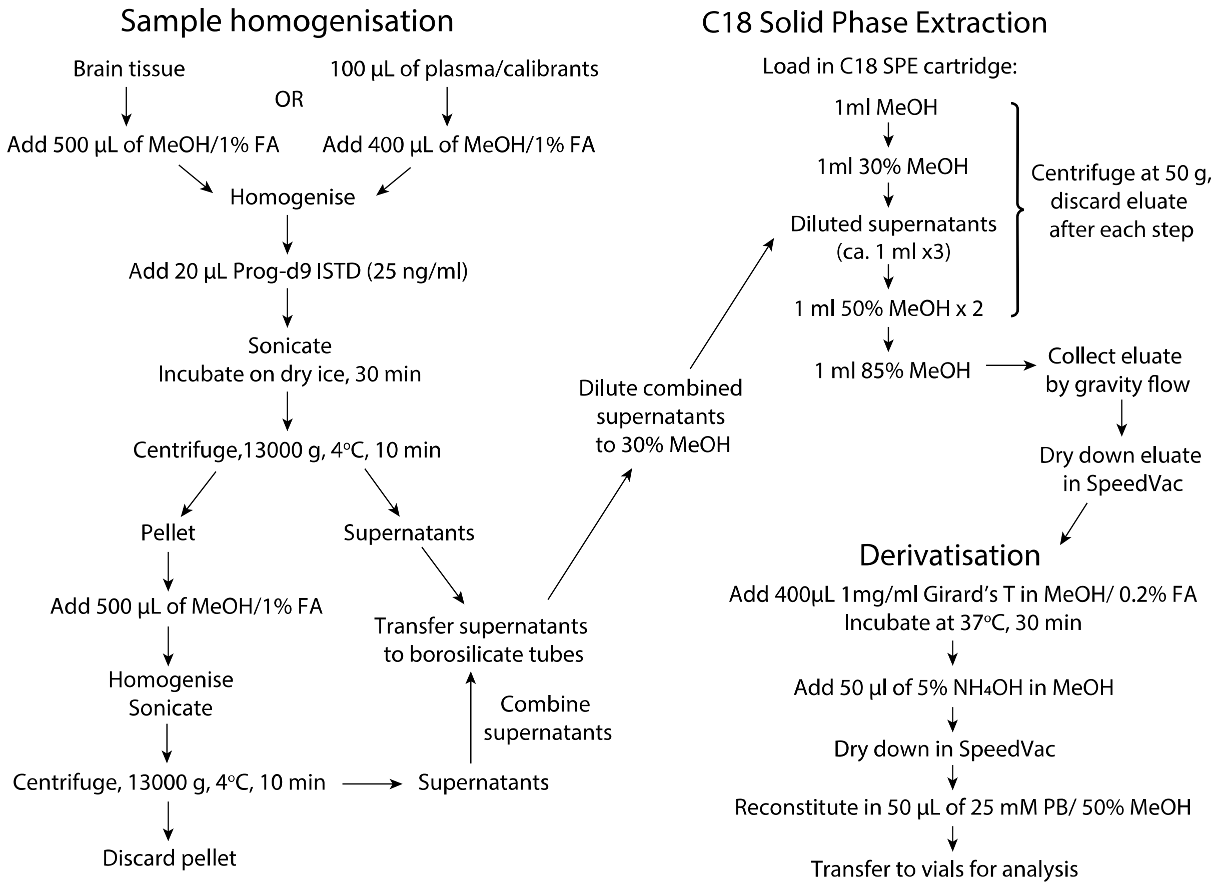

Supplementary Figure 2: Workflow for sample processing.

Brain and plasma samples were processed as above (*left column*), before solid phase extraction and derivatisation with Girard's T (*right column*).

Supplementary Table 1

LC parameters	
Injection volume	5µl; Partial loop injection
Flow rate	0.2 ml/min
Column temperature	40°C
Injection needle rinse	20% Methanol
Gradient:	
-4 min to 0 min	5% B
0 min	Sample injection
0 min to 1 min	5% B to 20% B
1 min to 6 min	45% B to 50% B
6 min to 11 min	50% B
11 min to 12 min	50% B to 100% B
12 min to 14 min	100% B
14 min to 15 min	100% to 5% B
Divert to waste	Before 2 min, after 12 min
Total run time	19 min
MS parameters	
Electrospray ionisation mode	Positive
Capillary current	-4500V
Nebulizer gas	Nitrogen; 16 psi
Dry gas	8 L/min
Dry temperature	150°C

Supplementary Table 1: Instrumentation set-up for liquid chromatography (*top panel*) and mass spectrometry (*bottom panel*)

Supplementary Table 2

Analyte	Molecular weight	Precursor ion (m/z)	Fragment ion (m/z)	Retention time (min)	Segment	Amplitude; Cut-Off
**Corticosterone**	346.5	460.2	401.1	3.5	1	117; 0.80
**Testosterone**	288.4	402.2	343.1	4.4	1	117; 0.80
**DOC**	330.5	444.2	385.1	4.6	1	120; 0.95
**DHDOC**	332.5	446.2	387.1	6.1	2	116; 0.80
**Pregnenolone**	316.5	430.2	371.1	6.3	2	116; 0.75
**Progesterone-d9**	323.5	437.5	368.3	8.2	3	116; 0.70
**Progesterone**	314.5	428.2	369.1	8.4	3	116; 0.75
**DHP**	316.5	430.2	371.1	8.6	3	116; 0.75
**Allopregnanolone**	318.5	432.2	373.1	10.4	4	116; 0.70

Supplementary Table 2: **Analytical parameters**

Analytical parameters for the target compounds and the internal standard (progesterone-d9). Derivatisation with Girard's T reagent produced a precursor ion $[M + G.T.]^+$, resulting in an addition of 114 Da to its absolute mass. Upon fragmentation by collision-induced fragmentation, a product ion with neutral loss of 59.1 Da (corresponding to the loss of the trimethylamine moiety) was produced, which was used for identification and quantification. Detection was carried out via multiple reaction monitoring, where no more than 3 analytes were monitored for each segment. Amplitude and cut-off parameters were optimised beforehand using a direct infusion method with a syringe pump.

Supplementary Table 3

Analyte	r^2	LOQ (pg/ml)	LOQ (pg, on column)	Calibration range (pg/ml)
**Corticosterone**	0.998	41	0.41	41 - 10000
**DOC**	0.998	41	0.41	41 - 10000
**DHDOC**	0.997	102.4	1.02	102.4 - 10000
**Progesterone**	0.998	256	2.56	256 - 25000
**DHP**	0.998	102.4	1.02	102.4 - 10000
**Pregnenolone**	0.998	102.4	1.02	102.4 - 10000
**Allopregnanolone**	0.996	256	2.56	256 - 25000
**Testosterone**	0.997	41	0.41	41 - 10000

Supplementary Table 3: Details of calibration curves

Calibration curves were constructed using the ratio of peak area of the target analyte and the peak area of progesterone-d9. Correlation coefficients (r^2) were calculated by weighted (1/x) regression analysis, obtained from the means of 6 calibration curves. The lower limit of quantification (LOQ) was defined as the concentration on the standard curve with a peak that is identifiable, discrete with a relative standard deviation of less than 20% and at least five times the signal of the blank. As 100µL of calibrant was used for sample processing, and only 5µL of the final reconstituted sample (50µL) was injected, the LOQ on-column (in pg) are also presented.

Supplementary Table 4

Analyte	QC level	Conc (pg/ml)	Recovery (%)	Intra-assay variability (%)	Inter-assay variability (%)	Accuracy (%)
**Corticosterone**	Low	500	74	16.9	24.9	111
	Medium	2000	71	19.5	22.9	104
	High	8000	84	14.4	22.0	98
**DOC**	Low	500	78	13.1	16.3	111
	Medium	2000	72	16.8	25.5	101
	High	8000	92	16.3	10.0	99
**DHDOC**	Low	500	95	16.5	15.1	140
	Medium	2000	82	6.6	24.9	113
	High	8000	108	9.0	7.8	112
**Progesterone**	Low	1250	71	17.9	13.7	126
	Medium	5000	80	5.5	6.5	98
	High	20000	83	8.0	4.1	104
**DHP**	Low	500	73	13.0	9.3	136
	Medium	2000	70	10.8	14.7	103
	High	8000	82	8.3	9.5	97
**Pregnenolone**	Low	500	88	17.6	24.8	110
	Medium	2000	81	8.4	16.6	89
	High	8000	95	12.5	9.6	93
**Allopregnanolone**	Low	1250	88	17.1	20.6	124
	Medium	5000	87	6.3	18.8	102
	High	20000	94	12.2	16.8	103
**Testosterone**	Low	500	95	18.5	16.5	132
	Medium	2000	105	7.7	8.8	106
	High	8000	106	10.4	10.3	109

Supplementary Table 4: **Assay performance characteristics**

Assay precision was examined using low, medium or high concentration (conc) of quality control standards (QC low, QC medium, QC high) in 4% BSA. 100 µl of QC standards were used and were processed as described in the methods, along with a set of standard calibrants. Intra-assay variability referred to the relative standard deviation (% RSD) of the values (pg/ml) obtained from four independent samples in a single run, against a single calibration curve. Inter-assay variability referred to the % RSD of four independent samples across four different runs, against four different calibration curves. Recovery was determined by comparing the peak areas for QC standards spiked before and after solid phase extraction (n=3). Accuracy was calculated using the following formula: '(obtained concentration/known concentration) x 100%', using the means of four independent samples. All samples were injected in duplicate and the means were used for all calculations.
